# Tumor necrosis factor alpha triggers mitochondrial dysfunction and ROS-driven cytokine amplification in bovine fibroblast-like synoviocytes

**DOI:** 10.3389/fvets.2026.1869964

**Published:** 2026-08-21

**Authors:** Natalia Andrea Godoy, Jan Gallastegui, Gabriela Velásquez, Stefanie Teuber, Sebastian Brauchi, John Quiroga, Pablo Alarcón, Gabriel Moran, María Angélica Hidalgo, Juan Hancke, Rafael Agustín Burgos

**Affiliations:** 1 Laboratory of Inflammation Pharmacology and Immunometabolism, Faculty of Veterinary Sciences, Institute of Pharmacology and Morphophysiology, Universidad Austral de Chile, Valdivia, Chile; 2 Laboratory of Cellular Biophysics, Faculty of Medicine, Institute of Physiology, Universidad Austral de Chile, Valdivia, Chile

**Keywords:** inflammation, mitochondria, reactive oxygen species, synoviocytes, TNF-*α*, bovine, fibroblast-like synoviocytes, lameness

## Abstract

Lameness in dairy cattle, driven by joint inflammation, involves fibroblast-like synoviocytes (FLS) that produce inflammatory mediators and reactive oxygen species (ROS). Tumor necrosis factor alpha (TNF-*α*) has been proposed as an early systemic inflammatory marker that precedes and accompanies the development of lameness in transition cows. TNF-*α* signaling promotes ROS production and amplifies the inflammatory response. However, the role of mitochondrial ROS (mtROS) in regulating pro-inflammatory gene expression, particularly in bovine joint inflammation, remains to be elucidated. In this study, we investigated the role of mtROS in TNF-*α*-stimulated bovine FLS. Using mitochondrial complex inhibitors, complexes I and III were identified as major contributors to mtROS generation. Antioxidants, such as N-acetyl-L-cysteine (NAC) and Mito-Tempo, suppress TNF-*α*-induced expression of IL-6, IL-8, and IL-1β, with NAC showing broader efficacy in inhibiting COX-2 expression, implicating both cytosolic and mitochondrial ROS in the inflammatory response. TNF-*α* increased the lactate/pyruvate ratio and the uptake of the fluorescent D-glucose analog 2-[N-(7-nitrobenz-2-oxa-1, 3-diazol-4-yl) amino]-2-deoxy-D-glucose (2-NBDG), suggesting an increase in glucose metabolism and a shift toward aerobic glycolysis. TNF-*α*-induced NOX2 expression was reduced by NAC and Mito-Tempo, thereby linking cytosolic ROS to inflammatory amplification. Mitochondrial networks dynamically shifted from a “networked” to “fragmented” phenotype after TNF-*α* exposure, accompanied by transient depolarization. Computational classification (UMAP/K-means) confirmed the morphological alterations. These findings demonstrate that mtROS and metabolic rewiring synergistically drive bovine synovial inflammation, highlighting mitochondrial dysfunction as a potential therapeutic target for joint inflammation-associated lameness.

## 1 Introduction

Lameness is a common and severely debilitating condition in dairy cattle that leads to decreased productivity, reduced reproductive efficiency, high culling rates, and compromised animal welfare (1, 2). Its etiology is multifactorial, including infectious diseases such as digital dermatitis and foot rot, as well as non-infectious factors such as trauma, poor management, genetic predisposition, nutritional imbalances, and digestive disorders such as ruminal D-lactic acidosis (3, 4). These factors can contribute to joint inflammation, which is a key pathological component in many cases of lameness.

Joint inflammation is the second most common cause of lameness in cattle (5), particularly in infectious diseases or conditions of metabolic imbalance that induce polysynovitis (6, 7). The synovial membrane is a critical site of inflammatory mediator production, and fibroblast-like synoviocytes (FLS), a resident mesenchymal cell type of the synovial lining, are recognized as active participants in inflammatory responses. Synovial fluid is secreted by the synovial membrane (synovium), which lines the articular capsule of joints. FLS are the predominant cell type in the synovial intima (8) and ensure the structural and physiological integrity of diarthrodial joints by regulating the synovial fluid composition and extracellular matrix of the joint lining (9). These cells produce and respond to cytokines and chemokines, contributing to the recruitment and activation of immune cells and perpetuating joint inflammation (10–12).

Tumor necrosis factor alpha (TNF-*α*) has been proposed as an early systemic inflammatory marker that precedes and accompanies the development of lameness in transition cows, with elevated serum and plasma TNF-*α* concentrations documented in animals with subclinical and chronic laminitis (13, 14). In a longitudinal trial, cows that became lame already showed a tendency for higher serum TNF-*α* concentrations 8 weeks pre-calving and displayed a significant health status × time interaction for this cytokine, positioning TNF-α as an inflammatory marker present in transition cows with lameness (13). Whether systemic elevations in TNF-*α* translate into increased articular concentrations remains to be determined in bovine species. However, TNF-α is among the most prominent cytokines detected in the synovial fluid of patients with rheumatoid arthritis and osteoarthritis, where it drives local amplification of the inflammatory response (15, 16). Supporting a local bovine-specific role, LPS stimulation of bovine hoof dermal cells increases TNF-*α* release, suggesting that this cytokine may contribute to the local inflammatory milieu in laminitis and other claw lesions associated with lameness (17).

In joints, FLSs express a wide range of pro-inflammatory molecules, including interleukin (IL)-1β, IL-6, IL-8, and cyclooxygenase-2 (COX-2), which are expressed in response to TNF-α and are involved in several inflammatory cascades (18–21). TNF-α signaling is associated with nuclear factor kappa B (NF-κB) activation and reactive oxygen species (ROS) production, which together orchestrate transcriptional responses that exacerbate inflammation (22, 23). Although the role of cytosolic ROS, primarily generated by NADPH oxidases (NOXs), has been extensively documented (24–27), the contribution of mitochondrial ROS (mtROS) in the regulation of pro-inflammatory gene expression is not well understood, particularly in the context of bovine joint inflammation (28, 29).

TNF-*α* can remodel cellular metabolism by upregulating glucose uptake and glycolytic flux via pathways involving glucose transporter 1 (GLUT1) and hypoxia-inducible factor 1 (HIF-1), which, in turn, alters the mitochondrial substrate supply and promotes increased mtROS production (30). These species serve as secondary messengers that regulate immune responses (31) and apoptotic pathways and participate in the controlled modulation of NF-κB, HIF-1, and other transcription factors (31, 32). However, excessive or dysregulated mtROS production can cause oxidative stress and tissue damage by promoting lipid peroxidation (33), mitochondrial DNA damage (34), and dysfunction of mitochondrial proteins (35); in spite of this, their relevance in veterinary contexts, including bovine joint disease, remains largely unexplored.

In this study, we elucidated the mechanism by which mtROS mediate TNF-*α*-driven inflammation in bovine FLS (bFLS). By integrating metabolic, molecular, and high-resolution imaging approaches, we showed that TNF-α (1) induces mtROS production primarily through complexes I and III of the electron transport chain; (2) amplifies pro-inflammatory gene expression (IL-6, IL-8, and COX-2) in an ROS-dependent manner; (3) increases aerobic glycolysis; and (4) triggers dynamic mitochondrial network fragmentation and transient inner membrane depolarization. In addition, our findings establish mtROS as a central regulator of synovial inflammation in cattle, linking metabolic dysfunction and mitochondrial dynamics to joint pathology, which is a previously unexplored axis in cattle.

## 2 Materials and methods

### 2.1 Cell culture

bFLS were isolated from the metacarpophalangeal joints of healthy Holstein Friesian heifers aged between 1 and 2 years, obtained at a local slaughterhouse under aseptic conditions, as previously described (36). The heifers were maintained on a free-range grazing system, supplemented with concentrates and forage. The experimental procedure was approved by the Institutional Committee for the Care and Use of Animals (CICUA) of the Universidad Austral de Chile (Protocol No. 576/2025). Cells were cultured in Dulbecco’s modified Eagle’s medium/Nutrient Mixture F12 (DMEM/F12; Thermo Fisher Scientific, Grand Island, NY, USA) supplemented with 10% fetal bovine serum (FBS; Biowest, Nuaillé, France), 1% penicillin/streptomycin (Sigma-Aldrich, St. Louis, MO, USA), 1 mM sodium pyruvate, and 25 mM HEPES, and maintained at 37 °C in a humidified 5% CO₂ atmosphere. Cells were used between passages 3 and 6 and plated 2 days prior to each experiment to reach ~70% confluence.

### 2.2 Mitochondrial ROS measurement

To evaluate mitochondrial superoxide production, 2.5 × 10^5^ cells were treated with 2.5 μM MitoSOX™ Red Mitochondrial Superoxide Indicator (Invitrogen™, Thermo Fisher Scientific, Carlsbad, CA, USA) at 37 °C for 30 min. When indicated, the cells were also incubated with rotenone (complex I inhibitor; Tocris Bioscience, Bristol, UK), antimycin A (complex III inhibitor; Sigma-Aldrich, St. Louis, MO, USA), or oligomycin A (complex V inhibitor; Santa Cruz Biotechnology, CA, USA). After incubation, the cells were detached using trypsin/EDTA, pelleted, resuspended in PBS, and analyzed using a BD FACSCanto II flow cytometer (BD Biosciences). The data were expressed as mean fluorescence intensity (MFI) and analyzed using FlowJo 7.6 software (TreeStar Inc., Ashland, OR, USA). In another set of experiments, 1 × 10^5^ cells/well in a 24-well cell culture plate (SPL Life Sciences, Pocheon, Korea) were loaded with 2.5 μM MitoSOX at 37 °C for 30 min. After washing twice with PBS, the cells were stimulated with 10 ng/mL TNF-*α* (Thermo Fisher Scientific, Waltham, MA, USA) and incubated at 37 °C for 120 min. MitoSOX fluorescence was assessed *in vivo* using a Leica DMi8 microscope (Leica, Wetzlar, Germany), with three random images taken per well at 40 × magnification. Finally, MitoSOX fluorescence intensity was quantified by ImageJ 1.53c software (National Institutes of Health, Bethesda, MD, USA).

### 2.3 Evaluation of pro-inflammatory gene expression using quantitative real-time RT-qPCR

To evaluate the role of ROS in inflammatory signaling, cells were pre-treated for 1 h with either 5 mM N-acetylcysteine (NAC) (Cayman Chemicals, USA), 1 μM Mito-Tempo (Cayman Chemical, Ann Arbor, MI, USA) or 1–10 μM diphenyleneiodonium chloride (DPI, an inhibitor of NOX enzymes; Sigma-Aldrich, St. Louis, MO, USA). Subsequently, the cells were stimulated with bovine TNF-α (10 ng/mL; Thermo Fisher Scientific, Waltham, MA, USA) for 6 h. The control group received vehicle (0.1% DMSO) treatment only. Total RNA was extracted using RNA Kit I (Omega Bio-Tek Inc., Norcross, GA, USA) and reverse-transcribed into cDNA with M-MLV Reverse Transcriptase (Promega, Madison, WI, USA). Quantitative PCR (RT-qPCR) was performed using Takyon® ROX SYBR Mastermix (Eurogentec, Seraing, Belgium) for IL-6 (F: TCCTGAAGCAAAAGATCGCA, R: CCACTCGTTTGAAGACTGC), IL-8 (F: AAACGAGGTCTGCCTAAACCC, R: TCTTGCTTCTCAGCTCTCTTCAC), pro-IL-1β (F: CCTCCGACGAGTTTCTGTGT, R: GCCAGCACCAGGGATTTTTG), NOX2 (F: AGATGCCTGGAAACTACCAAAGATA, R: TCACCACCTCGTAACTGAACAC), NOX4 (F: AGCCTGATGAACTTACCCAGAATAC, R: AGAAATCCAAAGCCAGGTCTGT), COX-2 (F: GCATAAGCTGCGCCTTTTCA, R: CAGGAACATGAGGCGGGTAG), normalized to RPS9 (F: CGCAAAACCTATGTGACCCC, R: CCTCCAGACCTCACGTTTGT) as the housekeeping gene in a StepOne Plus™ System (Applied Biosystems, Life Technologies, Foster City, CA, USA). The cycling conditions consisted of 40 cycles at 95 °C for 15 s, 60 °C for 30 s, and 72 °C for 30 s.

### 2.4 Quantification of IL-6 and IL-8 by enzyme-linked immunoassay

Cell culture supernatants were collected, centrifuged at 600 × g for 6 min to remove cellular debris, and stored until analysis. IL-6 and IL-8 protein concentrations were determined using a bovine-specific ELISA kit (catalog #ESS0029, Thermo Fisher Scientific and #3114-1A-6, Mabtech, Nacka, Sweden) according to the manufacturer’s instructions. Briefly, a 96-well plate pre-coated with capture antibody was blocked for 1 h with 4% BSA and 5% sucrose in PBS. Samples (100 μL) were added and incubated for 1–2 h, followed by sequential incubation with biotinylated detection antibody (1 h) and streptavidin-HRP (30 min), with two wash steps between each incubation. Color development was initiated with TMB substrate solution (20 min, protected from light) and stopped by addition of 0.16 M H₂SO₄. All steps were performed at room temperature. Absorbance was measured at 450 nm with correction at 550 nm using a Varioskan Flash spectrophotometer (Thermo Fisher Scientific).

### 2.5 Glucose uptake assay

To assess glucose uptake, bFLS were incubated overnight in low-glucose DMEM/F12 (5.5 mM) (Thermo Fisher Scientific, Grand Island, NY, USA). On the experimental day, the cells were washed and incubated in Krebs-Ringer-HEPES buffer (pH 7.4) containing 6 mM mannitol, 2 mM pyruvate, and 0.25% bovine serum albumin (BSA) for 1 h. Cells were then treated with TNF-*α* (10 ng/mL), insulin (50 μM), or vehicle in the presence of 100 μM of the non-metabolizable fluorescent glucose analog, 2-NBDG (Invitrogen™, Thermo Fisher Scientific, Carlsbad, CA, USA), at 37 °C for 30 min. Fluorescence intensity was measured before and after stimulation using flow cytometry (BD FACSCanto II). Data were analyzed using FlowJo 7.6 software (TreeStar Inc., Ashland, OR, USA).

### 2.6 Pyruvate and lactate profiling by GC–MS

Following a 1-h exposure to TNF-α (10 ng/mL) at 37 °C and 5% CO_2_ in sterile 21.5-cm2 plastic tissue culture plates (SPL Life Sciences, Pocheon-si, Korea), bFLS were rinsed twice with 0.9% NaCl and subsequently frozen in liquid nitrogen for 3 min. Subsequently, 1 mL of extraction buffer (comprising 37.5% vol/vol liquid chromatography/mass spectrometry (LC/MS)-grade acetonitrile, 37.5% vol/vol LC/MS-grade isopropanol, and 25% vol/vol LC/MS-grade water) containing 1 mM ribitol (# A5502, Sigma–Aldrich, St. Louis, MO, USA) was introduced into the cells. A rubber-tipped cell scraper was used to detach and collect the sample. The samples were vortexed for 2 min and centrifuged at 14,000 × g at 4 °C for 2 min. Subsequently, 450 μL of the supernatant was collected and completely dried under vacuum using a SpeedVac vacuum concentrator (Savant® SPD131DDA, Thermo Fisher Scientific, Waltham, MA, USA) at 45 °C for 90 min under 1.5 atm pressure. The dried samples were treated with 450 μL of wash buffer (50% vol/vol LC/MS-grade acetonitrile and 50% vol/vol LC/MS-grade water) and vortexed for 3 min. Following centrifugation at 14,000 × g at 4 °C for 2 min, the supernatants were evaporated to dryness in a SpeedVac concentrator at 45 °C for 90 min under 1.5 atm pressure. The dried samples were treated with 10 μL of a methoxyamine/pyridine hydrochloride solution (20 mg/mL) at 30 °C for 90 min. Next, 90 μL of MSTFA with 1% TMCS was added, along with 2 μL of a C8-C30 FAMEs mixture, which helps mark retention times. The mixture was incubated at 37 °C for 30 min with shaking. The samples were then placed in 250-μl glass vial inserts inside 2-ml glass vials with screw caps for analysis using gas chromatography–mass spectrometry (GC–MS). An Agilent 7890B GC system with a 5977 A mass-selective detector was used for the analysis. A 1 μL sample was injected in splitless mode into a guard column to separate the metabolites. The injector temperature was 250 °C, and helium gas flowed at 1 mL/min. The oven started at 60 °C and increased to 325 °C at 10 °C/min, taking 37.5 min in total. Mass spectra were collected in the 50–600 m/z range at 1.7 spectra/s with a 20 Hz scan rate, starting after a 5.9-min solvent delay. The MS ion source was maintained at 250 °C and the quadrupole at 150 °C. Data were processed using MS-DIAL v4.0, and analyses were performed using MetaboAnalyst 5.0. Lactate and pyruvate signals were normalized to ribitol, and the lactate/pyruvate ratio was estimated.

### 2.7 Mitochondrial morphology and membrane potential analysis in TNF-*α*–stimulated bFLS

bFLS cells were treated with TNF-α (10 ng/mL) for 0, 30, 60, 180, and 360 min. Thirty minutes before the endpoint, cells were incubated with 100 nM MitoTracker Red CMXRos (Thermo Fisher Scientific, Eugene, OR, USA) for 30 min. At the end point, cells were rinsed with fresh media before imaging. Live-cell images were acquired using a Nikon Eclipse Ti-S inverted epifluorescence microscope (Nikon, Japan) with a 543 nm excitation laser and a 605 ± 55 nm emission filter. Thirty-frame XYT stacks were acquired at 3 Hz. To maximize spatial resolution for mitochondrial morphology analysis, images were acquired using a 60X, 1.4NA objective. A single cell was analyzed per field of view.

#### 2.7.1 Image processing and analysis

The images were preprocessed using FIJI/ImageJ 1.53c software (National Institutes of Health, Bethesda, MD, USA) using a 2-pixel median filter, 50-pixel rolling paraboloid background subtraction, and average intensity projection. Images were further enhanced using unsharp masking and segmented using the Phansalkar local thresholding method (radius = 15 pixels). Morphological parameters, including mitochondrial area, number of branches, total branch length, and branch junctions, were extracted using the Mitochondria Analyzer plugin (37).

#### 2.7.2 Machine learning-based mitochondrial classification

The morphological parameters obtained via Mitochondria Analyzer were analyzed with a semiautomatic ML-based tool implemented in Python 3.x with scikit-learn, umap-learn, pandas, seaborn, and matplotlib. The extracted morphological parameters (i.e., mitochondrial area, branch number, total branch length, and branch junction number) were represented in a four-dimensional feature space and reduced to two dimensions using Uniform Manifold Approximation and Projection (UMAP; n_neighbors = 40, min_dist = 0.1), followed by K-means clustering. UMAP dimensionality reduction was performed over the complete dataset of 163 individual cells, regardless of the treatment. To evaluate clustering robustness, dimensionality reduction was repeated using different parameter combinations, yielding consistent segregation patterns across runs. The optimal number of clusters was determined using the elbow method, Davies–Bouldin index, and statistical separation of the morphological feature distributions. Cluster identity was assigned ex-post, based on the average morphological characteristics of the cells within each cluster. Clusters displaying higher branch number, branch junction number, and total branch length were classified as networked, whereas clusters displaying lower connectivity, reduced branching and lower mitochondrial area were classified as fragmented.

#### 2.7.3 Functional analysis of mitochondrial membrane potential

To evaluate the mitochondrial membrane potential (Δψm), bFLS were incubated with 25 nM tetramethylrhodamine ethyl ester (TMRE) for 30 min under standard culture conditions, as previously described. Time-lapse fluorescence recordings were acquired at 1 Hz for 7 min. Cells were acutely stimulated with TNF-*α* (10 ng/mL) for 60 s, followed by treatment with FCCP (1 μM) for 360 s to induce full depolarization. For chronic exposure, cells were pre-treated with TNF-α (10 ng/mL) for 360 min before TMRE analysis. Fluorescence data were processed using a custom rolling-window local fractional fluorescence algorithm to calculate log₂ (F/F₀). Maximum intensity projections of the first five frames were used to extract mitochondrial network masks and morphological parameters to classify the mitochondrial morphology using UMAP.

### 2.8 Statistical analysis

Data normality was assessed using the Shapiro–Wilk test, and variance homogeneity was assessed using Levene’s test. Morphological analyses were performed over 163 individual bFLS cells, obtained from three independent bovine donors and inspected across five independent experimental days. Each experimental day corresponded to cells derived from a single donor preparation. Morphological parameters were analyzed separately for each mitochondrial cluster. Depending on the data structure, one-way analysis of variance (ANOVA) was used for parametric comparisons, whereas Kruskal-Wallis tests were used for non-parametric distributions. Results were considered statistically significant at *p* < 0.05. All analyses were performed using GraphPad Prism or a Python-based statistical software package.

## 3 Results

### 3.1 Mitochondrial ROS generation driven by electron transport chain

**Figure 1:**
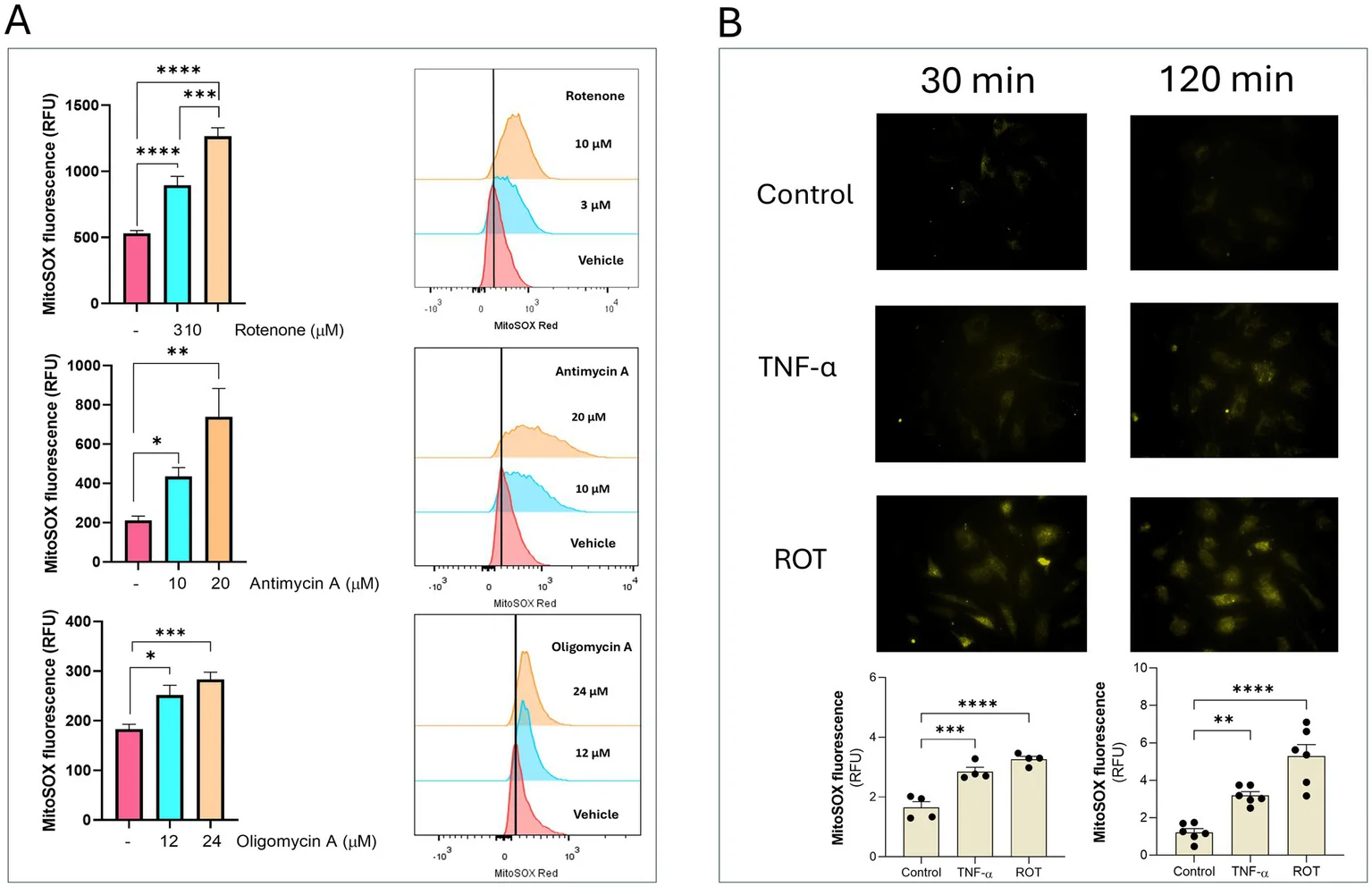
TNF-*α* induces mitochondrial ROS (mtROS) production in bFLS. **(A)** bFLS were treated with different concentrations of rotenone, antimycin A, and oligomycin A or vehicle for 30 min in the presence of 2.5 μM MitoSOX. After cell detachment, the cells were analyzed by flow cytometry, *N* = 4. **(B)** bFLS were stimulated with TNF-α (10 ng/mL) for 30 and 120 min in the presence of 2.5 μM MitoSOX. The cells were analyzed *in vivo* using fluorescence microscopy, *N* = 4–6. Bar graphs represent the mean fluorescence ± SEM. Representative histograms and pictures are shown for each experiment. *p < 0.05, **p < 0.01, ***p < 0.001, ****p < 0.0001.

To determine the origin of mtROS in bFLS, 1.5 × 10^5^ cells were treated with inhibitors of mitochondrial complexes I (3 and 10 μM rotenone), III (10 and 20 μM antimycin A), and V (12 and 24 μM oligomycin A) (Figure 1A). Flow cytometry analysis using the MitoSOX probe demonstrated a significant increase in fluorescence intensity following all three treatments, indicating elevated mitochondrial superoxide levels. The effect was dose-dependent and statistically significant compared to that of the vehicle (*p* < 0.05 to *p* < 0.0001), suggesting that electron transport chain complexes I and III contribute to mtROS generation in bFLS. In addition, oligomycin A increased the fluorescence of MitoSOX Red, supporting the potential role of complex V in mtROS production in bFLS. MitoSOX signal induced by TNF-*α* was below the sensitivity threshold of flow cytometry under our experimental conditions and was therefore quantified by fluorescence microscopy (Figure 1B). TNF-*α* stimulation induced a significant time-dependent increase in MitoSOX fluorescence in bFLS (30 min: *p* < 0.001; 120 min: *p* < 0.01 *vs*. unstimulated control), confirming that TNF-α promotes mitochondrial superoxide generation. Rotenone produced a significantly greater MitoSOX signal (*p* < 0.0001 *vs*. TNF-α), consistent with ETC complex I as a source of TNF-*α*-driven mtROS in bFLS.

### 3.2 Antioxidants mitigate TNF-α-induced pro-inflammatory gene expression

**Figure 2:**
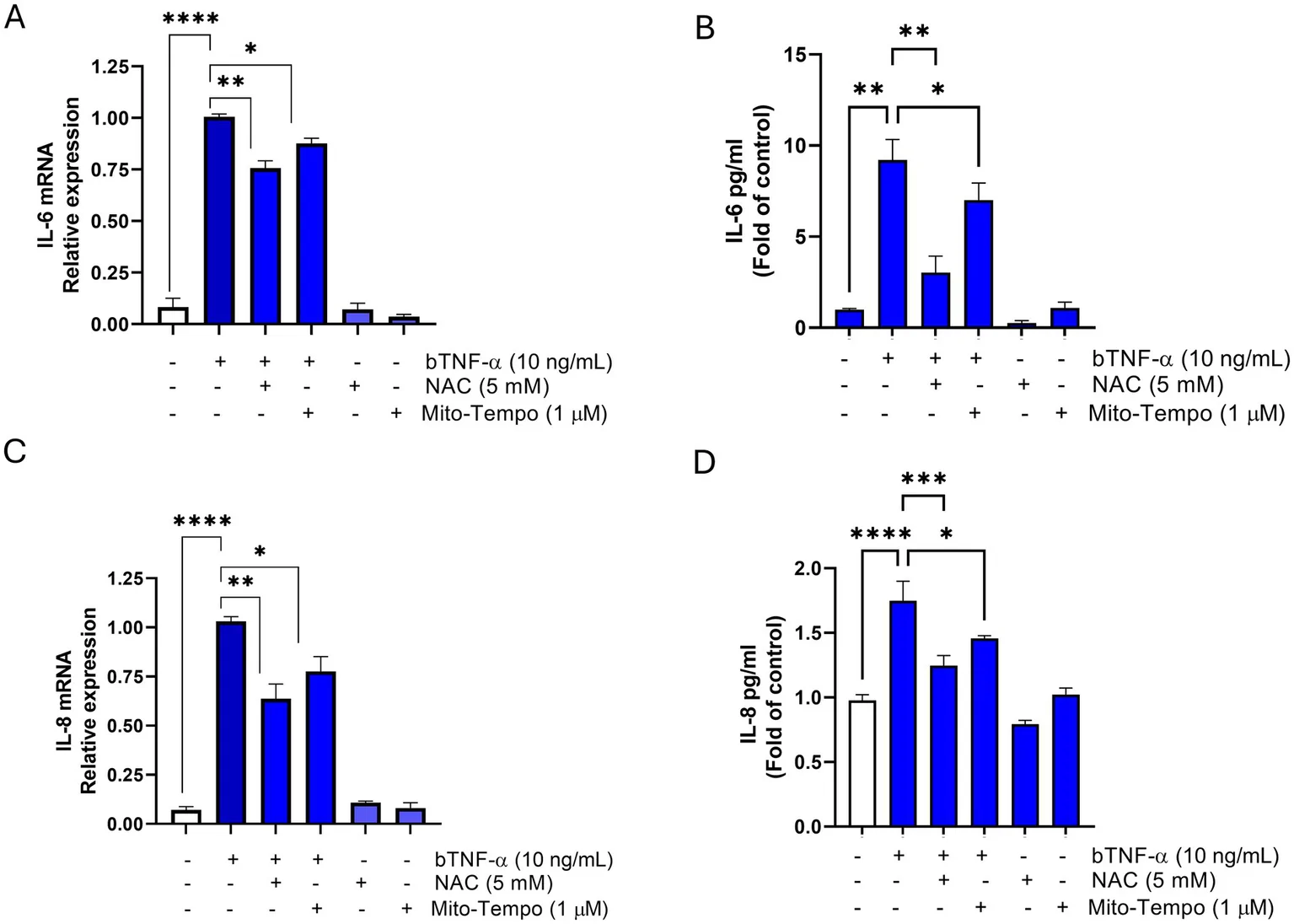
NAC and mito-tempo partially decrease the production of IL-6 and IL-8 induced by TNF-α. bFLS were pre-treated with either NAC (5 mM) or Mito-Tempo (1 μM) for 1 h, followed by TNF-α exposure (10 ng/mL) for 6 h. RNA was extracted and used for cDNA synthesis, and qPCR was conducted to measure IL-6 **(A)** and IL-8 **(C)** mRNA expression. In addition, the secretion of IL-6 **(B)** and IL-8 **(D)** was measured in supernatants by ELISA. Data are presented as mean ± SEM. *N* = 4. *p < 0.05, **p < 0.01, ***p < 0.001, and ****p < 0.0001.

Pretreatment with NAC (5 mM) or the mitochondria-targeted antioxidant Mito-Tempo (1 μM) prior to TNF-α stimulation significantly reduced the expression and secretion of IL-6 (Figures 2A,B) and IL-8 (Figures 2C,D) in bFLS. These results also indicate that ROS from mitochondria and other cellular sources contribute with the expression of pro-inflammatory cytokines induced by TNF-*α*. In addition, we also observed that the increase in pro-IL-1β and COX-2 mRNA expression induced by TNF-α was inhibited by NAC; however, Mito-Tempo only reduced the expression of pro-IL-1β (Supplementary Figure S1). Moreover, we demonstrated that NAC also attenuated rotenone-induced mtROS production, as measured by MitoSOX fluorescence intensity (Supplementary Figure S2).

### 3.3 Changes in pyruvate and lactate levels and glucose uptake induced by TNF-*α*

**Figure 3:**
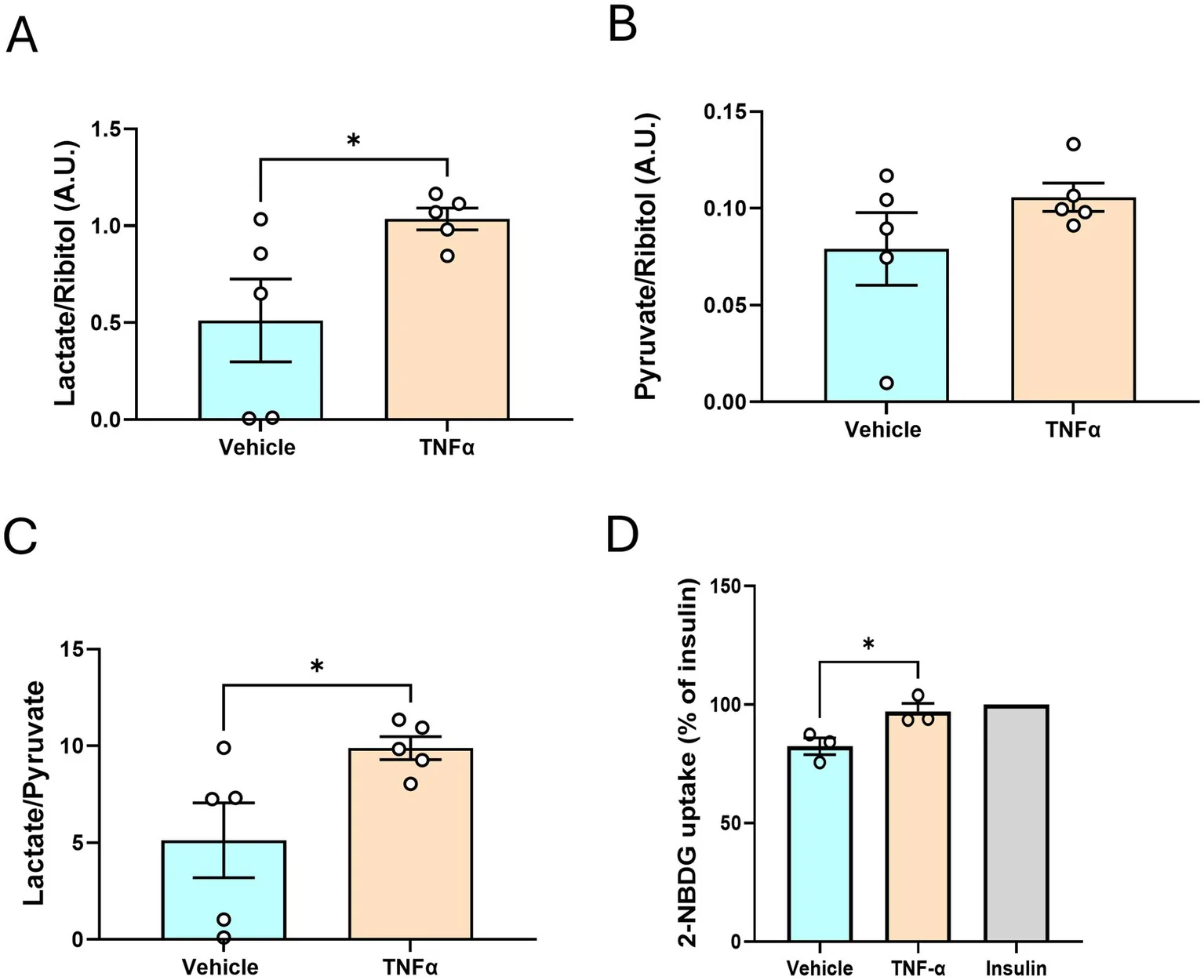
TNF-α increases lactate accumulation and glucose uptake in bFLS. **(A–C)** bFLS were stimulated with TNF-α (10 ng/mL) or vehicle for 1 h. Relative abundance of lactate **(A)** and pyruvate **(B)**, normalized to ribitol (internal standard), was determined by GC–MS. **(C)** Lactate/pyruvate ratio calculated from GC–MS data, reflecting the cellular redox state and glycolytic flux. **(D)** bFLS were treated with vehicle, TNF-α (10 ng/mL), or insulin (50 μM) for 30 min. Glucose uptake was measured as 2-NBDG fluorescence by flow cytometry and expressed as a percentage of the insulin-stimulated response. Data are presented as mean ± SEM. *N* = 4–5. *p < 0.05.

TNF-α treatment induced a significant increase in lactate and lactate/pyruvate ratios (Figures 3A–C). Notably, the pattern of metabolite changes reflects a profound alteration in the cellular redox state, where the accumulation of lactate underscores a compensatory mechanism to sustain ATP production during the inflammatory response. Consistent with metabolic reprogramming, TNF-α stimulation significantly increased glucose uptake in bFLS (Figure 3D), with insulin showing the maximum response. This result indicates an enhanced glycolytic flux that could compensate for the altered mitochondrial substrate utilization. Moreover, this mechanism can be used as a fuel for ROS production via the NADPH oxidase. In addition, this metabolic rewiring creates conditions conducive to increased mtROS production from a functionally active electron transport chain that operates under altered substrate availability and redox conditions.

### 3.4 TNF-*α* upregulates NOX2 mRNA expression

**Figure 4:**
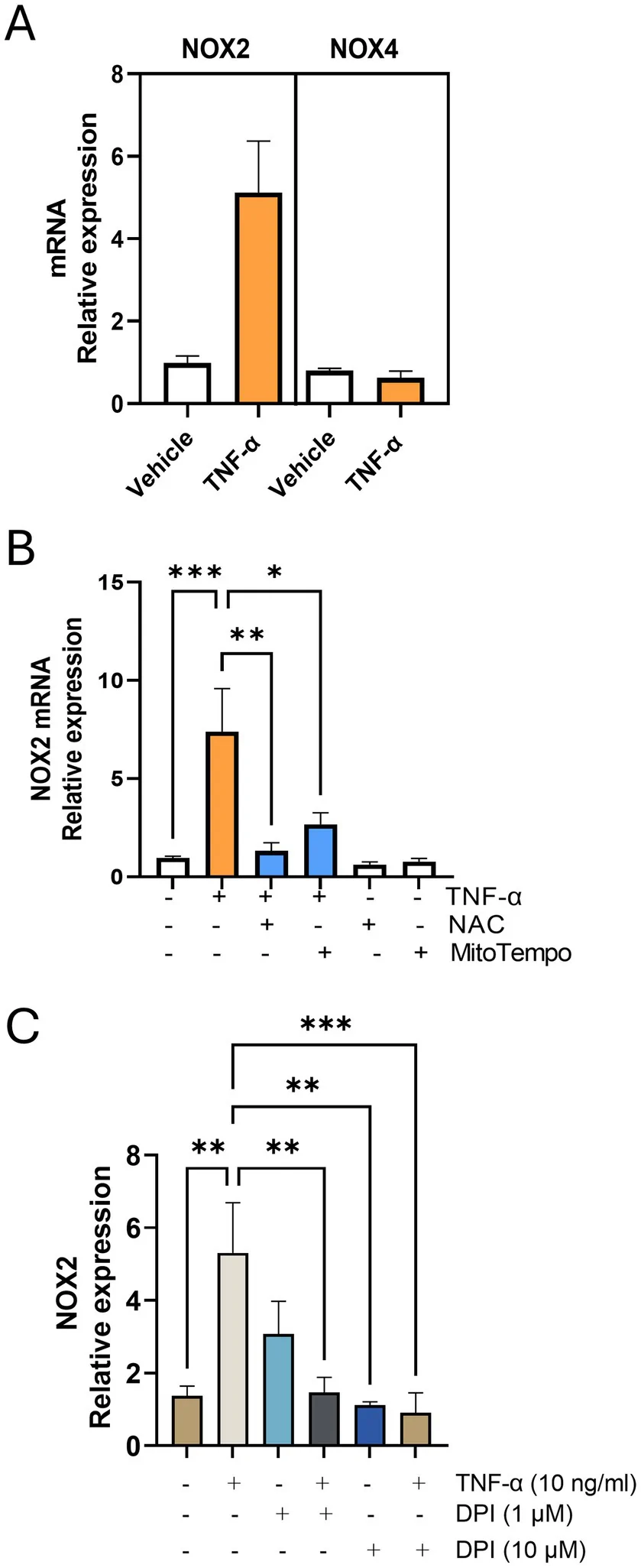
TNF-α upregulates NOX2 expression in bFLS, which is suppressed by antioxidants and NADPH oxidase inhibition. **(A)** bFLS were stimulated with TNF-α (10 ng/mL) or vehicle for 6 h. The mRNA expression of NOX2 and NOX4 was evaluated by RT-qPCR. **(B)** bFLS were pretreated for 1 h with NAC (5 mM) or Mito-Tempo (1 μM) prior to TNF-α stimulation (10 ng/mL) for 6 h. The mRNA expression of NOX2 was evaluated by RT-qPCR. **(C)** bFLS were pretreated with DPI at 1 or 10 μM for 1 h and then stimulated with 10 ng/mL TNF-α for 6 h. The mRNA expression of NOX2 was evaluated by RT-qPCR. Data are presented as mean ± SEM. *N* = 4. *p < 0.05, **p < 0.01, ***p < 0.001.

To explore the contribution of NOX enzymes in ROS production, we first assessed the mRNA expression of NOX2 and NOX4, enzymes associated with superoxide generation in the cytoplasm, using RT-qPCR. The analysis showed that TNF-α significantly upregulated the expression of NOX2, but not of NOX4 (Figure 4A), in TNF-α-stimulated bFLS (*p* < 0.05). Interestingly, preincubation of bFLS with NAC and Mito-Tempo significantly abrogated the TNF-*α*-induced expression of NOX2 (Figure 4B). Furthermore, DPI reduced significantly the expression of NOX2 (Figure 4C). These findings support the hypothesis that both mitochondrial and non-mitochondrial ROS sources are involved in amplifying inflammatory responses.

### 3.5 UMAP-based classification reveals two distinct mitochondrial network states and dynamic shifts in mitochondrial morphology over time

**Figure 5:**
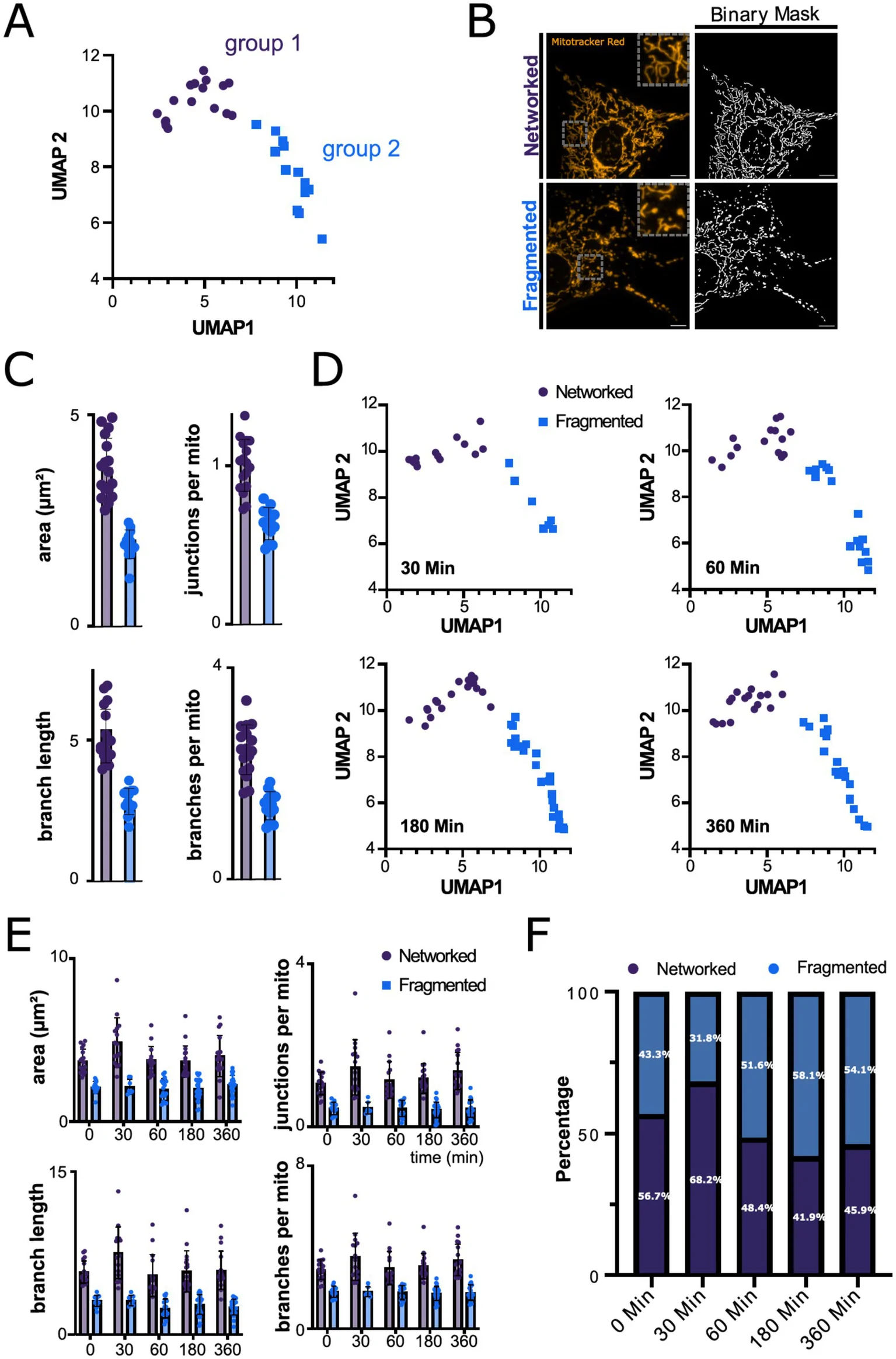
UMAP-based classification of bFLS mitochondrial morphologies under bTNF-α stimulation. **(A)** UMAP projections of untreated bFLS cells morphological parameters. Two primary groups were identified; ex-post re-inspection of individual cells allows their classification in networked and fragmented. 30 cells from 10 cultures obtained from 3 donors were recorded in 4 independent days of experiments. **(B)** Representative images of cells displaying networked and fragmented mitochondrial phenotypes. Binary masks were used for morphological quantification, and the classification was determined via UMAP. **(C)** Morphological features observed in untreated cells that were used for dimensionality reduction. These include mean area (μm^2^), branches per mitochondrion, total branch length (μm), and branch junction per mitochondrion. **(D)** UMAP projections of morphological parameters extracted from bFLS cells exposed to bTNF-α for 30, 60, 180 and 360 min. **(E)** Time-resolved comparison of individual morphological parameters grouped by classification (networked or fragmented). All four features showed significant differences between morphological states (networked versus fragmented; Kruskal–Wallis or ANOVA, *p* < 0.01), with branch junctions per mitochondrion showing the most pronounced effect (*p* < 0.001). T30: 24 cells from 7 cultures, recorded in 3 independent days of experiments. T60: 34 cells from 10 cultures, recorded in 5 independent days of experiments. T180: 45 cells from 12 cultures, recorded in 4 independent days of experiments. T360: 35 cells from 7 cultures, recorded in 3 independent days of experiments. **(F)** Proportions of networked versus fragmented cells at each time point post–bTNF-α exposure.

It has been established that changes in metabolite utilization and redox conditions affect the architecture of the mitochondrial network (38, 39). Therefore, we explored whether TNF-*α* exposure induces mitochondrial fragmentation in cultured bFLS. To this end, we first performed a computational analysis of synoviocyte mitochondrial networks, followed by the classification of each individual cell via UMAP dimensionality reduction and K-means clustering. From the grouping analysis, we identified two primary clusters in the untreated cells (Figure 5A). Further inspection showed that the clusters corresponded well to networked and fragmented phenotypes (ANOVA and Kruskal-Wallis, *p* < 0.01; branch junctions, *p* = 4.52 × 10^−6^; Figures 5B,C; Supplementary Figure S3).

The networked phenotype exhibited a larger mitochondrial area, greater number of branches, longer branch lengths, and more junctions per mitochondrion. In contrast, fragmented mitochondria were smaller, with fewer branches and shorter lengths (Figure 5C; Supplementary Figure S3). Morphological analysis of chronic TNF-*α* incubation (0–360 min post-TNF-α) revealed that the proportion of cells with fragmented mitochondria changed over time. At shorter time windows (up to 60 min), the grouping structure changes and recovers at longer incubation times (Figure 5D). Regardless, the grouping strategy showed strong consistency at every time point analyzed (Figure 5E). When analyzing the proportion of the two phenotypes, we observed an early increase in the number of mitochondria in the network, which were reshaped into a more fragmented shape over time (Figure 5F). Thus, our analysis revealed dynamic remodeling of the mitochondrial networks in response to TNF-*α*, potentially reflecting a physiological response to a pro-inflammatory insult as well as an adaptive response over longer periods of continuous exposure.

### 3.6 TNF-*α* induces transient mitochondrial depolarization

**Figure 6:**
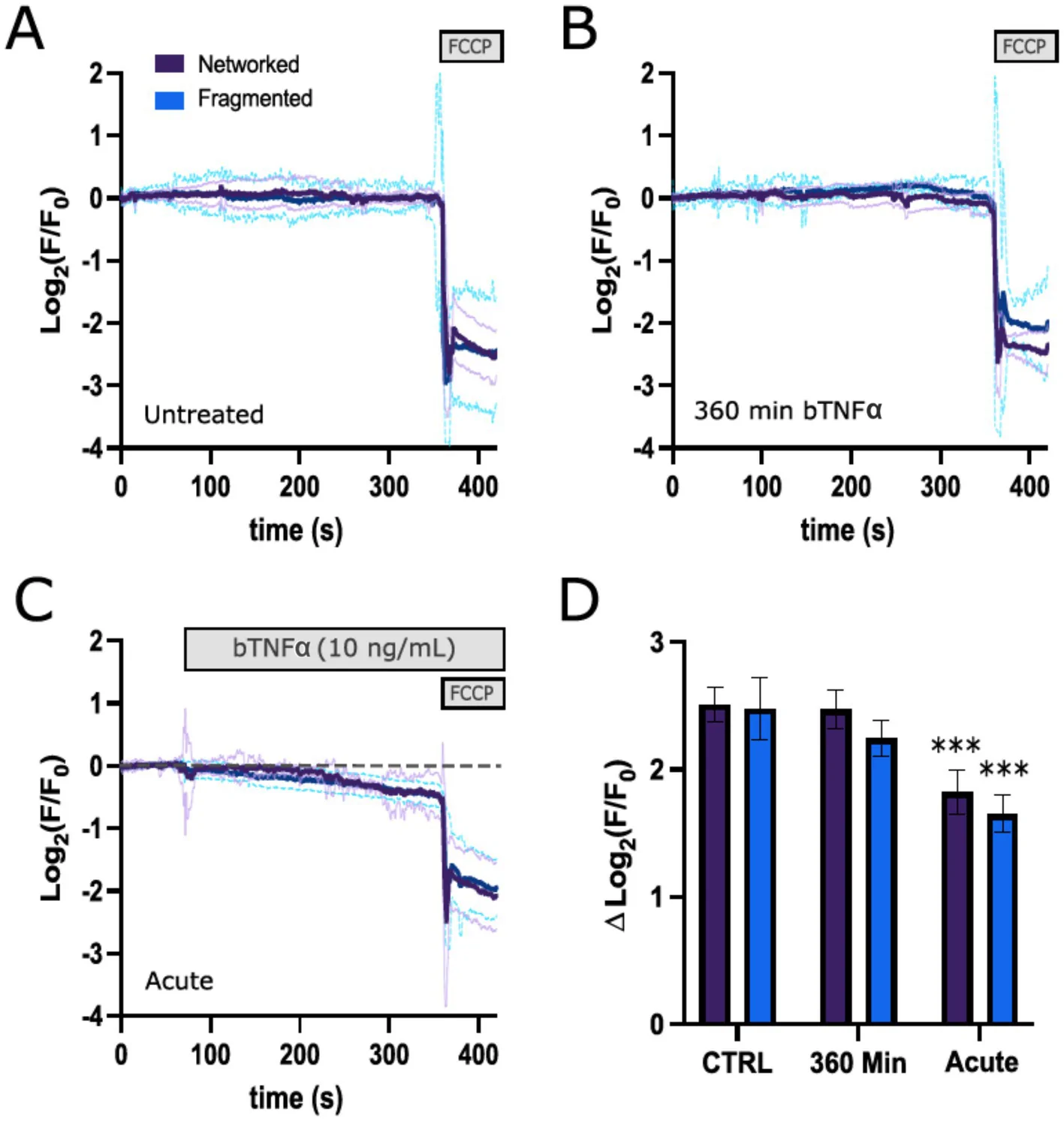
Functional analysis under bTNFα stimulation of bFLS cells mitochondrial membrane potential. Representative traces of TMRE mitochondrial voltage recordings (ΔΨm) in response to the mitochondrial uncoupler FCCP (indicated) in three conditions of bTNFα stimulation (10 ng/mL; indicated). Changes are presented in logarithmic scale to facilitate comparisons between positive and negative deflections. **(A)** Untreated cells. **(B)** 360 min TNF-α incubation. **(C)** Acute exposure to 10 ng/mL TNF-α. **(D)** Steady-state ΔΨm potential values (calculated as: 220–320 s baseline minus FCCP-treated final 20 s) grouped by morphology and condition. Acute 10 ng/mL TNF-α treatment significantly reduced membrane potential in both networked and fragmented cells compared to control and 360-min treatments (Welch’s *t*-tests, *p* < 0.05).

Both morphological and metabolic changes have been shown to correlate with changes in the electrical response of the inner mitochondrial membrane (38). The observed response profile, characterized by early reshaping followed by compensatory mechanisms, suggests a direct effect on mitochondrial physiology, which is intimately linked to the Δψm. To determine whether acute TNF-*α* affects Δψm, we loaded the cells with TMRE and obtained time courses via live-cell imaging. We observed that the electrical response to FCCP treatment indicated that the average membrane potential was similar between the fragmented and networked groups (Figure 6A). In agreement with the morphological data, prolonged exposure of cells (360 min) elicited a Δψm comparable to that of the control (Figure 6B). In contrast, acute TNF-*α* treatment (10 ng/mL) resulted in a significant decrease in Δψm in both networked and fragmented mitochondrial populations compared to the untreated control (Welch’s t-test, *p* < 0.05; Figures 6C,D). Overall, our results show that transient depolarization coincides with an early adaptive response to mitochondrial stress, triggering both architectural and metabolic changes upon exposure to TNF-*α*.

## 4 Discussion

This study demonstrates that TNF-α stimulation of bFLS leads to significant mitochondrial dysfunction and ROS-driven cytokine amplification mediated through both mitochondrial and cytosolic pathways. These findings expand our current understanding by highlighting the role of mtROS in pro-inflammatory gene expression during bovine joint inflammation. Overall, these results suggest that mtROS contribute to the oxidant environment induced by TNF-α and pro-inflammatory cytokine expression in bFLS. In support of this, Mito-Tempo decreases mtROS generated by TNF-α, thereby blunting p38/JNK activation and IL-6/IL-8 expression in macrophages and endothelial cells (40, 41). Mutations in TNFR1 have been shown to elevate mtROS levels in human monocytes and peripheral blood mononuclear cells (PBMCs) from patients with factor receptor-associated periodic syndrome (TRAPS). Targeting mtROS with the mitochondria-specific antioxidant MitoQ (an analog of Mito-Tempo) nearly abolishes the LPS-induced increase in IL-6, TNF-α, CXCL8/IL-8, and IL-10 in both patient and healthy donor PBMCs, without affecting IRF3-dependent genes such as IFN-*β* and CCL5/RANTES (40). In humans, FLSs from patients with rheumatoid arthritis co-cultured with Mito-Tempo-treated PBMCs displayed significantly reduced mRNA levels of IL-1β, IL-6, IL-8, TNF-α, and other inflammatory cytokines, supporting the role of mtROS in paracrine inflammatory signaling (42). The antioxidant effect of NAC also inhibits the sustained phosphorylation of JNK and p38 in TNFR1 mutant mouse embryonic fibroblasts stimulated with LPS (40), while in endothelial cells, it reduces TNF-α-mediated IL-8 production via p38 MAPK (43). NAC reduces intracellular ROS primarily by serving as a cysteine precursor for glutathione (GSH) biosynthesis, thereby replenishing intracellular antioxidant capacity, and secondarily through direct thiol-mediated scavenging of reactive species (44, 45). Emerging evidence suggests that the antioxidant effect of NAC does not primarily rely on direct radical scavenging but rather on its bioconversion within the mitochondria into persulfide sulfur species. This process involves the enzymatic activities of 3-mercaptopyruvate sulfurtransferase (MST) and sulfide:quinone oxidoreductase (SQR), which catalyze the formation of reactive sulfur intermediates that serve as immediate effectors of NAC’s redox-protective actions in the mitochondria (46).

Mitochondria are the primary consumers of oxygen and generate ROS through electron leakage from the electron transport chain (ETC). Complexes I and III are the main sources of superoxide, which under basal conditions represents 0.2–2.0% of reduced oxygen and is rapidly converted to H₂O₂ by superoxide dismutases (47, 48). In bFLS, modulation of complexes I, III, and V with rotenone, antimycin-A, or oligomycin, respectively, increased mtROS. Oligomycin-induced mtROS elevation occurs through mitochondrial hyperpolarization and prolonged electron lifetime in complexes I and III, facilitating superoxide formation, and has been shown to increase IL-8 levels in rat synovial tissue (49, 50). Rotenone, antimycin-A, or oligomycin can activate NLRP3 inflammasomes, MAPK/NF-κB signaling, and IL-1β, IL-6, and IL-8 expression (41, 51–58). These findings underscore the relevance of mtROS as both initiators and amplifiers of inflammatory signaling in bFLS.

The mtROS response is further amplified by TNF-*α*–induced upregulation of the NADPH oxidase isoform NOX2, suggesting a synergistic pro-oxidant environment that reinforces inflammatory gene expression (59, 60). NOX2 is normally expressed at low levels but is upregulated by pro-inflammatory cytokines, priming NADPH oxidase via ERK/p38-mediated phosphorylation of p47phox and enhancing ROS production upon secondary stimulation. These ROS sustain NF-κB and MAPK signaling, potentiating IL-1β/IL-6–driven responses (61, 62). In HEK293 cells, TNF-*α* increases ROS via NOX2 and activates JNK/ERK in a redox-dependent manner (63), and NOX2-derived ROS have also been observed in human umbilical vein endothelial cells upon Angiopoietin-1 stimulation (64). NAC and Mito-Tempo reduced NOX2 expression in TNF-*α*-induced bFLS, supporting the cooperative role of mtROS and NOX2-derived ROS in amplifying inflammation.

We previously demonstrated that TNF-α increases the expression of HIF1-α, GLUT1, LDHA, PDK1, and IL-6 and stabilizes HIF1-α. We proposed that these metabolic and inflammatory changes were dependent on HIF-1, since pharmacological inhibition with YC-1 reduced the overexpression of HIF-1α, GLUT1, and PDK1, as well as the expression and secretion of IL-6 (65).

Instead, TNF-α promotes a glycolytic shift, characterized by increased glucose uptake, elevated GLUT1 expression, and upregulation of glycolysis-related enzymes, including HK2, PFKFB3, and LDHA, as well as PDK1, a kinase that promotes lactate production by inhibiting pyruvate entry into the TCA cycle, resulting in elevated lactate production (30, 66, 67). The increase in lactate, lactate/pyruvate ratio, and 2-NBDG uptake observed in the present study is consistent with a glycolytic metabolic shift induced by TNF-α. This interpretation is supported by our previous work in the same bFLS model demonstrating that TNF-α induces bona fide metabolic reprogramming, including increased LDHA expression and activity, elevated extracellular lactate, reduced TCA cycle intermediate flux as determined by D-glucose-^13^C6 isotope tracing, and glycolysis-dependent expression of proinflammatory mediators (67). This shift toward glycolysis likely meets both bioenergetic and biosynthetic demands during inflammation, whereas limited mitochondrial respiration may favor ROS overproduction via the pentose phosphate pathway, creating a pro-inflammatory metabolic state that sustains cytokine expression and synovial activation.

TNF-α–induced metabolic rewiring in bFLS favors glycolysis without affecting oxidative metabolism, reminiscent of the Warburg effect, providing energy, biosynthetic precursors, and ROS necessary to sustain inflammation. These metabolic adaptations may also influence epigenetic regulation, redox-sensitive transcription factors, and immune cell crosstalk within the joints (30, 66, 68).

Machine learning analyses (UMAP and K-means clustering) categorized bFLS mitochondria into networked or fragmented forms. In our model, TNF-α induced an early increase in the mitochondrial network, which was followed by fragmentation that correlated with increased mtROS. The partial recovery of the normal proportion between the two morphologies is indicative of adaptive mitochondrial responses that help maintain bioenergetic and redox homeostasis (38, 68). Interventions targeting the fission–fusion balance may provide opportunities to modulate inflammatory responses in synovial tissue, preventing excessive ROS production and cytokine release.

Oxidative damage to mitochondrial components is associated with rapid mitochondrial fragmentation and the transition from elongated tubules to punctate organelles (69). Fragmented mitochondria contribute to increased ROS leakage, impaired oxidative phosphorylation, and apoptosis-resistant proinflammatory phenotypes (70). Mitochondrial dynamics, the continuous balance between fission and fusion, are central to maintaining mitochondrial function. Fission allows the segregation of damaged mitochondrial segments, facilitating their removal by mitophagy; however, excessive fission leads to fragmented mitochondria with elevated ROS production, impaired energy metabolism, and activation of inflammatory pathways such as NLRP3 and NF-κB (71, 72). Conversely, fusion promotes the mixing of mitochondrial contents, dilutes damaged components, maintains the mitochondrial membrane potential, and supports ATP production. Fusion contributes to the maintenance of tubular mitochondrial networks that are more efficient in oxidative phosphorylation and exhibit lower ROS leakage, supporting anti-inflammatory and reparative cellular states (73, 74). The temporal coordination of fission and fusion may be critical in determining whether synoviocytes adapt to or undergo sustained inflammatory activation.

Transient mitochondrial depolarization, measured using TMRE, was normalized by 360 min, suggesting the activation of repair mechanisms or the selection of resilient mitochondrial subpopulations (75, 76). However, even transient depolarization may lead to the release of mitochondrial damage-associated molecular patterns (DAMPs), such as mtDNA and formyl peptides, which can activate pattern recognition receptors, further amplifying inflammation and potentially causing additional mitochondrial damage (77, 78). These DAMPs can influence neighboring synoviocytes, chondrocytes, and infiltrating immune cells, thereby integrating mitochondrial stress into paracrine and autocrine inflammatory loops.

Overall, these results highlight the role of mtROS in amplifying the inflammatory responses of bFLS. The interplay between metabolic reprogramming, mitochondrial dynamics, and ROS generation reveals multiple potential therapeutic intervention points. Targeted antioxidant strategies that combine mitochondrial protection with the inhibition of NOX- and mtROS-dependent pathways may effectively disrupt this pathogenic loop. In parallel, modulation of glycolytic flux, mitochondrial fission–fusion balance, and damage-associated molecular pattern (DAMP) signaling could provide complementary approaches to limit chronic synovial inflammation.

This study has limitations inherent to its *in vitro* design, which used isolated bovine fibroblast-like synoviocytes. Although this reductionist approach enabled the precise dissection of TNF-*α*–induced mitochondrial and redox signaling pathways, it does not fully capture the complex cellular, metabolic, and biomechanical interactions present within the inflamed joint *in vivo*. Accordingly, extrapolation of these findings to whole-joint pathology or clinical lameness should be made with caution and requires further in vivo validation studies. Therefore, future studies should investigate whether metabolic and redox crosstalk among synoviocytes, chondrocytes, and immune cells modulates these pathways in vivo, providing a more comprehensive understanding of joint inflammation.

In conclusion, TNF-α drives mitochondrial dysfunction in bFLS through a coordinated mechanism involving mitochondrial superoxide generation, NOX2 upregulation, altered glucose metabolism, and biphasic mitochondrial remodeling. The identification of a mtROS–NOX2 amplification loop as a previously uncharacterized feature of TNF-α signaling in bovine synoviocytes advances mechanistic understanding of synovitis and supports the therapeutic targeting of ROS pathways in lameness-associated joint inflammation.

## Data Availability

The original contributions presented in the study are included in the article/Supplementary material, further inquiries can be directed to the corresponding author.
