# Supplementary material for: Tumor necrosis factor alpha triggers mitochondrial dysfunction and ROS-driven cytokine amplification in bovine fibroblast-like synoviocytes
Source: Front Vet Sci. 2026 Aug 21;13:1869964. doi: 10.3389/fvets.2026.1869964 (PMC13541457; doi:10.3389/fvets.2026.1869964)
Supplement: Supplementary file 1 [file Data_Sheet_1.PDF]

Supplementary Material

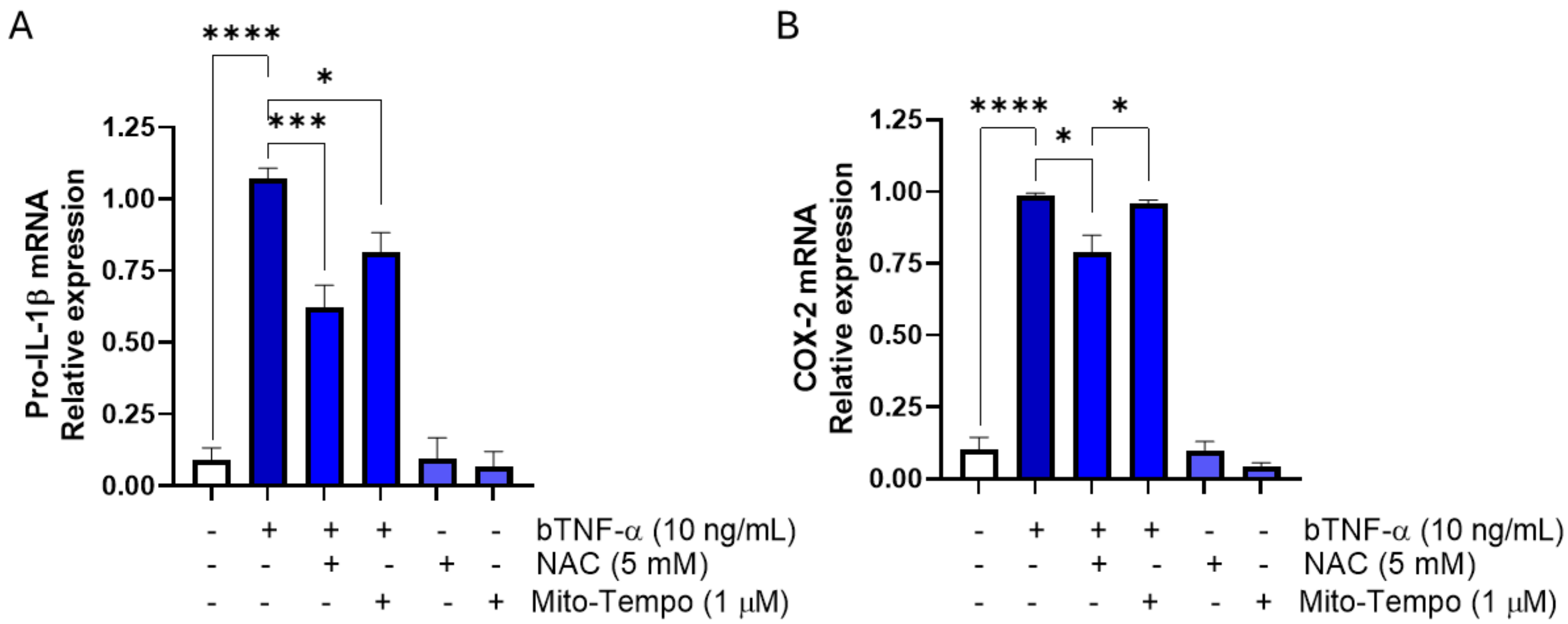

Supplementary Figure S1. NAC and Mito-Tempo partially decrease the expression of pro-IL-1β and COX-2 induced by TNF-α. bFLS were pre-treated with either NAC (5 mM) or Mito-Tempo (1 μM) for 1 h, followed by TNF-α exposure (10 ng/ml) for 6 h. RNA was extracted and used for cDNA synthesis, and qPCR was conducted to measure pro-IL-1β **(A)** and COX-2 **(B)** mRNA expression. Data are presented as mean ± SEM. N = 4 * $P < 0.05$, *** $P < 0.001$, and **** $P < 0.0001$.

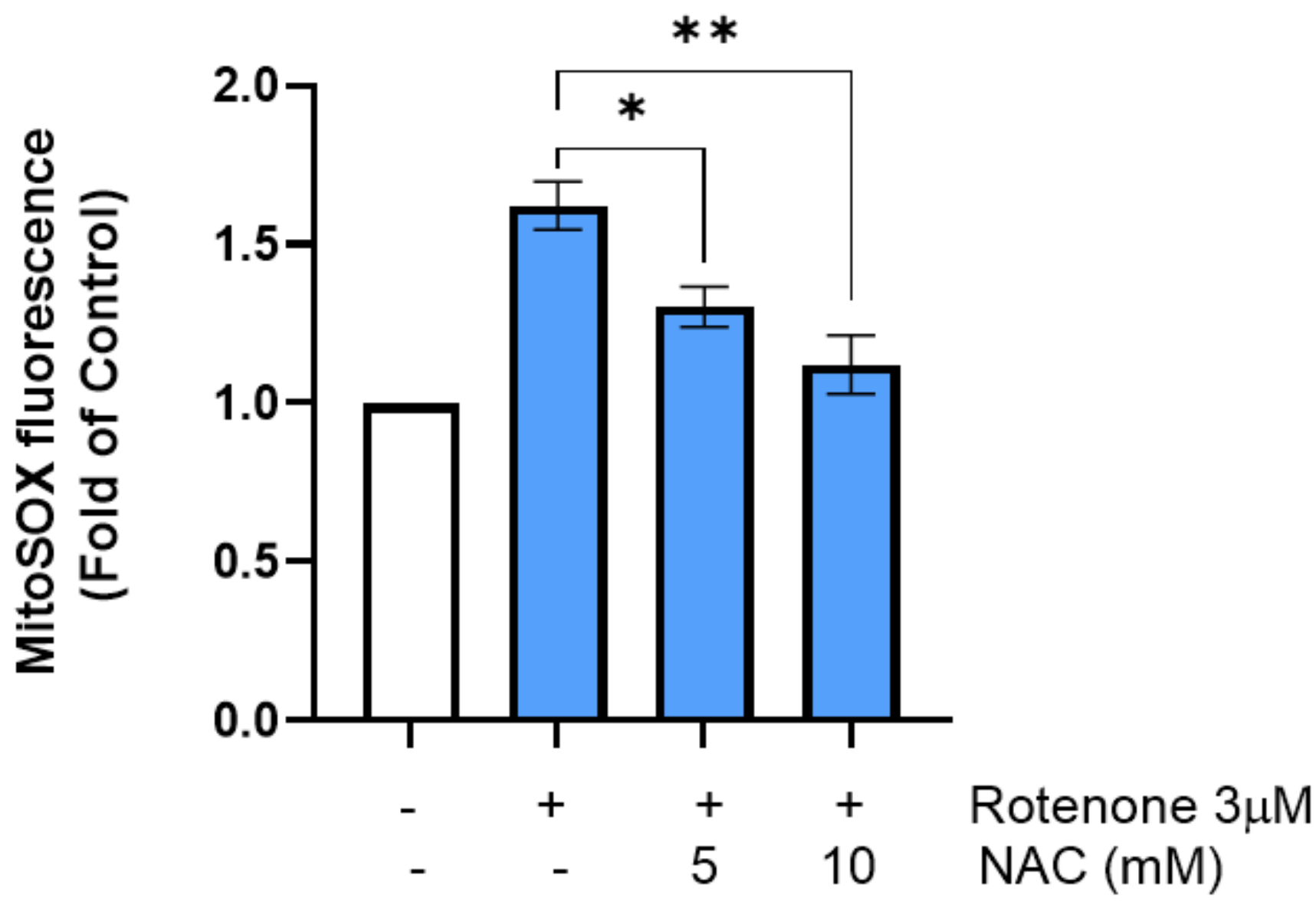

Supplementary Figure S2. NAC reduces mtROS production induced by rotenone. bFLS were pre-treated with NAC (5 and 10 mM) for 1 h, followed by rotenone exposure (3 μM) for 30 min, and analyzed by flow cytometry. Data are presented as mean ± SEM, n = 4, * $P < 0.05$; ** $P < 0.01$.

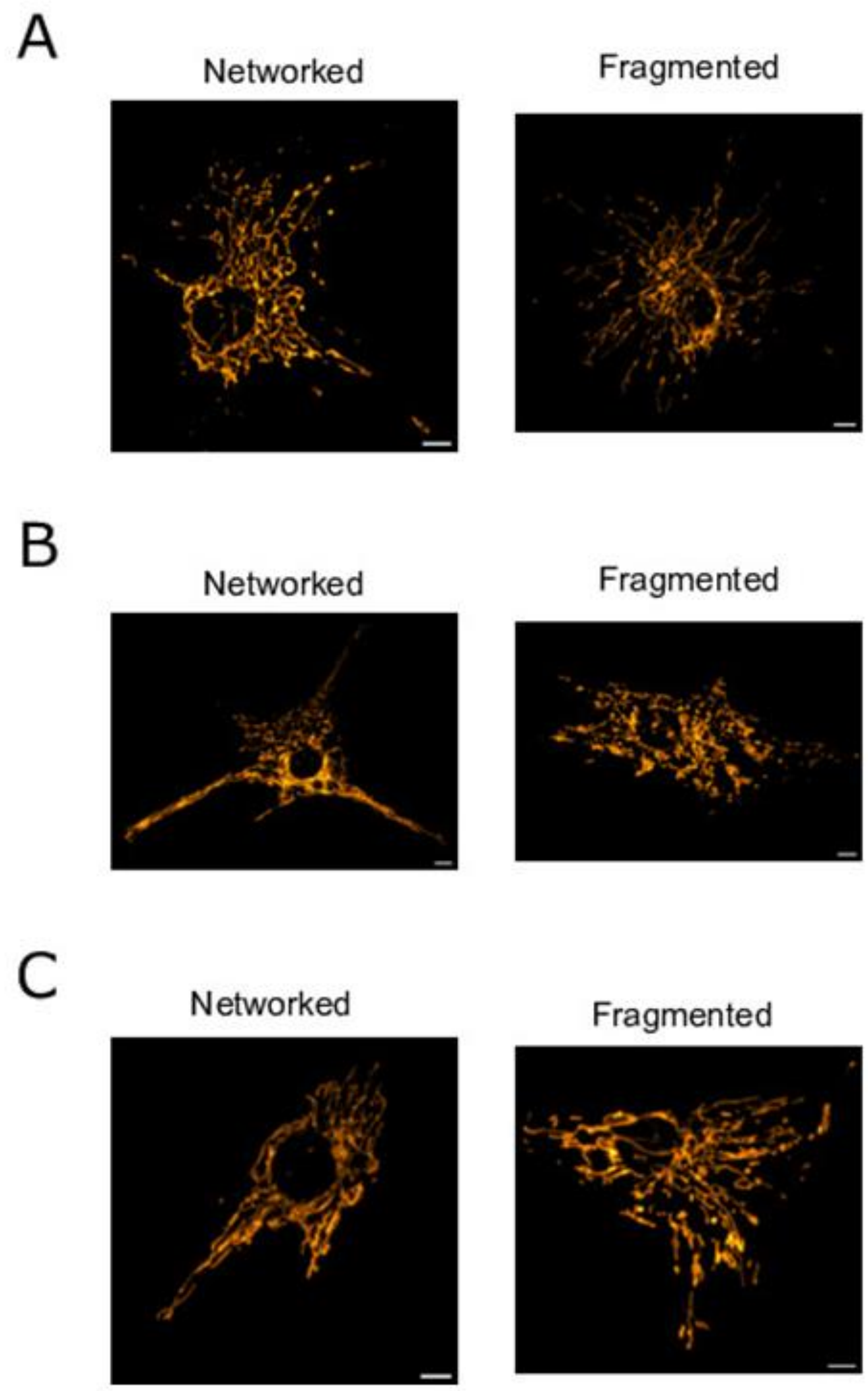

Supplementary Figure S3. Representative images of UMAP classified mitochondrial phenotypes in bFLS. (A) Untreated conditions. **(B)** 360 minutes of bTNF-α incubation. **(C)** Acute treatment.
